# Expression Profiling after Prolonged Experimental Febrile Seizures in Mice Suggests Structural Remodeling in the Hippocampus

**DOI:** 10.1371/journal.pone.0145247

**Published:** 2015-12-18

**Authors:** Bart C. Jongbloets, Koen L. I. van Gassen, Anne A. Kan, Anneke H. O. Olde Engberink, Marina de Wit, Inge G. Wolterink-Donselaar, Marian J. A. Groot Koerkamp, Onno van Nieuwenhuizen, Frank C. P. Holstege, Pierre N. E. de Graan

**Affiliations:** 1 Brain Center Rudolf Magnus, Department of Translational Neuroscience, University Medical Center Utrecht, Utrecht, the Netherlands; 2 Molecular Cancer Research, Division Biomedical Genetics, University Medical Center Utrecht, Utrecht, the Netherlands; 3 Brain Center Rudolf Magnus, Department of Pediatric Neurology, University Medical Center Utrecht, Utrecht, the Netherlands; Radboud University Nijmegen, NETHERLANDS

## Abstract

Febrile seizures are the most prevalent type of seizures among children up to 5 years of age (2–4% of Western-European children). Complex febrile seizures are associated with an increased risk to develop temporal lobe epilepsy. To investigate short- and long-term effects of experimental febrile seizures (eFS), we induced eFS in highly febrile convulsion-susceptible C57BL/6J mice at post-natal day 10 by exposure to hyperthermia (HT) and compared them to normotherm-exposed (NT) mice. We detected structural re-organization in the hippocampus 14 days after eFS. To identify molecular candidates, which entrain this structural re-organization, we investigated temporal changes in mRNA expression profiles eFS 1 hour to 56 days after eFS. We identified 931 regulated genes and profiled several candidates using in situ hybridization and histology at 3 and 14 days after eFS. This is the first study to report genome-wide transcriptome analysis after eFS in mice. We identify temporal regulation of multiple processes, such as stress-, immune- and inflammatory responses, glia activation, glutamate-glutamine cycle and myelination. Identification of the short- and long-term changes after eFS is important to elucidate the mechanisms contributing to epileptogenesis.

## Introduction

Febrile seizures are the most prevalent type of seizures among children up to 5 years of age (2–4% of Western-European children) [1]. Although simple febrile seizures are generally benign, complex and especially prolonged febrile seizures, lasting longer than 10 minutes with recurring episodes, are thought to contribute to the development of epilepsy (epileptogenesis). Retrospective studies amongst temporal lobe epilepsy (TLE) patients show a higher incidence of complex or prolonged febrile seizures during childhood compared to other types of epilepsy, and hence have been suggested to be a precipitating or aggravating factor in the development of TLE [2–5]. In addition, a series of prospective studies indicate that children with prolonged febrile seizures are at risk of hippocampal injury and development of hippocampal sclerosis, a common pathology among TLE patients [6,7]. Prolonged febrile seizures together with risk factors such as a family history of epilepsy, age of onset and underlying neurological abnormality, increase the risk of epilepsy later in life [8,9]. Genetic predisposition to febrile seizure susceptibility in human and mice shows that the molecular make-up of the brain contributes to the occurrence and impact of febrile seizures [10–12]. Animal models are highly suitable to study the relation between febrile seizures and epilepsy, and provide opportunity to monitor the underlying process of epileptogenesis.

Several animal models for febrile seizures have been developed, in which a temperature controlled chamber is used to trigger hyperthermia and subsequently the development of febrile seizures in rats and mice [13–15]. The development and onset of febrile seizures can be determined by behavioral and by electroencephalographic (EEG) criteria [13,14]. Studies on the effects of experimentally induced prolonged febrile seizures (eFS) in rat and mouse indicate structural and functional changes in temporal lobe brain areas such as the hippocampus and amygdala [15–20]. Hallmarks of such changes in the hippocampus after eFS are mild mossy fiber sprouting and ectopic migration of neonatally generated granule cells in the dentate gyrus, as well as CA1 neuronal hyper-excitability and a shift in activity-dependent synaptic plasticity [15,17,18,21]. Control experiments elegantly showed that these hippocampal changes are eFS-induced and not merely a consequence of the induced hyperthermia [13,15,21,22]. The molecular processes mediating these eFS-induced structural and functional changes are still largely unresolved. In resemblance with humans, eFS elicited in rodents are followed by a latent period until the first signs of spontaneous epileptiform activity as observed among 35–68% of the exposed animals [15,22,23]. The molecular changes that occur during the latent phase which eventually lead to spontaneous epileptiform activity are still largely unknown.

To investigate short- and long-term changes after eFS, we induced eFS in highly febrile convulsion susceptible C57BL/6J mice (post-natal age 10, P10) by warm-air induced hyperthermia (HT)[14]. We compared hippocampal mRNA expression profiles of eFS and normothermic control mice at various times (up to 56 days) after eFS induction. Subsequently we validated several regulated genes by qPCR and immunocytochemistry.

## Materials and Methods

### Ethics statement

All experiments conformed to institutional guidelines of the University Medical Center Utrecht, approved by the Animal Ethics Committee of Utrecht University, and were conducted in agreement with Dutch laws (Wet op de Dierproeven, 1996) and European regulations (Guideline 86/609/EEC).

### Animals

Mice were bred from pairs of C57BL/6J mice (Jackson Laboratories, ME, USA) and raised by their mothers. Birth of new litter was daily checked and the date of birth was defined as postnatal day zero (P0). Between P0-P2, large litters (>6 pups) were reduced to a maximum of six pups with a balanced male/female ratio where possible. Pups were weaned on P21 and housed maximum four per cage. Animals were kept in a controlled 12h light-dark cycle with a temperature of 22 ± 1°C and were given unrestricted access to food (211 RMH-TM diet; Hope Farms, Woerden, NL) and water. Animals were housed in transparent Plexiglas cages with wood-chip bedding and tissue for nest building. Animal reaching the human end-point, defined by < 20% weight loss over 2 days, do not receive care from mother, or loss of grooming before the end of the experiment were deceased from the study. All animals at the end of the experiment or reaching the human endpoint were sacrificed by cervical decapitation.

### Warm-air induced prolonged febrile seizures

To induce eFS by hyperthermia (HT), mice were exposed to a temperature-regulated laminar stream of warm air (41–48°C) to quickly raise the core body temperature as previously described [14]. Briefly, body weight was determined in P10 C57BL/6J mice. At least 30 minutes before the HT procedure, mice were injected subcutaneously with temperature sensitive transponders (IPTT-300, PLexx, Elst, NL). Two temperature chambers were used, one for age-, weight-, and litter-matched normothermic control mice (NT; core body temperature maintained at 35°C) and one for HT (defined as core body temperature > 39°C) mice. Chamber temperature was maintained at 32°C for NT mice and at 47°C for HT mice. Mice were placed one by one on the floor of the preheated chambers. Core body temperature was measured every 20 seconds by wireless transporter readout (WRS-6006/6007, Plexx). HT was typically reached within 2.5 minutes. After core temperature of the HT mice had reached 42°C, air temperature was adjusted to maintain a core body temperature between 41.5°C and 42°C. Time and temperature data was logged automatically on a computer (DASHost v1.0, Plexx). HT was maintained for 30 minutes. Then mice were partly submerged in water of room temperature to quickly normalize core body temperature and returned to their mother. NT littermates were simultaneous treated as HT mice, except that their core body temperature was kept at 35°C. The observer scored the stages of behavior and seizures during the experiment, as previously described [14]. Only mice showing continued febrile seizures during at least 17.5 minutes (97.3% of all HT-exposed mice) were used in the experiment.

### Tissue collection and sectioning

To explore the temporal relationship of structural changes after eFS, mice were sacrificed by decapitation and brains were removed at three days and fourteen days after eFS. The brains were fresh frozen and stored at -80°C for radioactive *in situ* hybridization or incubated overnight (ON) in 4% paraformaldehyde (PFA) in phosphate-buffered saline (PBS) at 4°C for immunohistochemistry.

### Cryo-sectioning for radioactive *in situ* hybridization

Frozen brains were sectioned on a cryostat (Leica CM3050, Rijswijk, NL). Coronal sections (16 μm) containing the hippocampus were collected on Superfrost slides (Merzel, Germany) by 3 sequential sections per slide until a whole cross section was made of the hippocampus. Sections for protein and mRNA quantification were sampled between Bregma -1.46 mm and -2.54 mm. Slides were stored at -80°C until used for *in situ* hybridization.

### Paraffin-sectioning for immunohistochemistry

After overnight incubation with 4% PFA in PBS, brains were transferred to 70% Ethanol and subsequently embedded in paraffin. Paraffin-embedded brains were sectioned on a microtome (Leica). Coronal sections (7 μm) containing the hippocampus were collected on Superfrost slides (Merzel) by 3 sequential sections per slide until a whole cross section was made of the hippocampus. Slides were stored at room temperature until used for immunohistochemistry.

### Immunohistochemistry

To determine eFS-induced changes in protein expression, immunohistochemistry (IHC) was performed on coronal sections (7 μm) from animals fourteen days after eFS (NT *n* = 10, HT *n* = 10) using antibodies raised against CNP (mouse, 1:24,000, Sigma Aldrich, USA) and NF (medium polypeptide, mouse, 1:600, DSHB, USA). Using serial dilutions, the optimal working concentration of each antibody was determined. After dehydration, all sections were subjected to antigen retrieval using microwave treatment (7 minutes 650W, 5 minutes 350W) in 0.01 M Na^+^Citrate pH 6.0. Peroxidase activity was blocked by treatment with 0.3% H_2_O_2_ in PBS-0.2% triton-X (PBS-t) for 30 minutes, followed by blocking of non-specific antigen binding with 4% horse normal serum in PBS-t at 37°C for 30 minutes. Sections were incubated with primary antibodies in PBS-t overnight at 4°C. Next, sections were incubated with secondary antibody biotinylated horse anti-mouse (1:400, Brunschwig Chemia, Amsterdam, NL) for 1 hour at room temperature, followed by antigen visualization using avidin-biotin method (Vectastain ABC Elite Kit, Vector Laboratories, USA) with 3,3’-diaminobenzidinetetrachloride (DAB) as the chromogen (Sigma Aldrich). No immunostaining was observed when the protocol was completed without primary antibodies (data not shown). All sections stained for NF or CNP were imaged in one session using a light microscope (Axiophot, Zeiss, Germany), with exposure and light intensity kept constant. Photographs were analyzed using the image-processing program ImageJ (version 1.43, US National Institute of Health, MD, USA). Regions of interest were analyzed bilaterally in the stratum lucidum by an observer blind for treatment group. Expression levels were calculated by gray-scale value inversion and normalization [24]. Mean intensity values of both hemispheres at 3 sections per animal were calculated and used for statistical analysis. Background intensities were measured in the stratum lacunosum. Average intensities per animal were grouped per condition and plotted in Tukey-style boxplots. Pair-wise comparison of average DAB intensity values was tested using the Wilcoxon signed-rank test.

### Hippocampal dissection and RNA isolation

One hour, three, fourteen, and fifty-six days after HT animals were sacrificed by decapitation. Brains were quickly removed and hippocampal tissue was dissected, collected and stored at -80°C. Total RNA was isolated, purified and checked for quality as described [25].

### Microarray analysis

To identify critical and functional mediator genes during epileptogenesis, acute, short-, and long-term effects of prolonged febrile seizures on gene expression were investigated in whole hippocampal samples. Samples were taken from C57BL/6J animals one hour (HT *n* = 8, NT *n* = 8), three days (HT *n* = 6, NT *n* = 6), fourteen days (HT *n* = 6, NT *n* = 6), and fifty-six days (HT *n* = 6, NT *n* = 6) after HT. Two-channel oligonucleotide microarray analysis was performed as described [26,27]. Briefly, cDNA from 2 μg total RNA was synthesized using a T7 oligo(dT)24VN primer (Ambion, Cambridgeshire, UK). The T7 Megascript kit (Ambion) was used for cRNA synthesis and its quality analyzed. Cy3 or Cy5 fluorophores (GE Healthcare Europe, Diegem, BE) were coupled to 2μg NT and HT cRNA. The degree of label incorporation was monitored and hybridizations were set up with 1,500 ng, 2–3% Cy5/Cy3 labeled cRNA per channel. Hybridization was performed with a NT and HT sample on the same chip, including a dye-swap (technical replicate). Just before hybridizing, HT and NT sample were mixed and fragmented (AM8740, Austin, TX, USA). The mouse Array-Ready oligo set (version 3, Operon Biotechnologies, Cologne, DE) was printed on UltraGAPS slides (Corning, Schiphol-Rijk, NL). Probe sequences from this array were re-annotated by BLAST-searching against genomebuild version 71_37 at Ensembl. Slides were washed by hand and scanned on an Agilent G2565BA scanner at 100% laser power, 30% PMT. After image analysis using Imagene 5.6.1 (BioDiscovery), Loess normalization was performed [26] per printtip on mean spot-intensities. Gene-specific dye bias was corrected by a within-set estimate [27]. Data were further analyzed by MAANOVA [28], modeling sample, array and dye effects in a fixed effect analysis. *P*-values were determined by a permutation F2-test, in which residuals were shuffled 10,000 times globally. Gene probes with *P* < 0.05 after false discovery rate determination (FDR according to Benjamini-Hochberg, BH) were considered significantly changed. In cases of multiple probes per gene, values from the lowest *P*-value and most regulated probe were used. For all time points a fold change (Fc) cutoff of log2(Fc) = < - 0.125, or > 0.125 was set, all probes with Ensembl annotation for genesymbol and description, excluding annotations for pseudo- and predicted- genes, were used to produce the significant gene list.

The data discussed in this publication have been deposited in NCBI's Gene Expression Omnibus [29] and are accessible through GEO Series accession number GSE66762.

### Quantitative RT-PCR

cDNA was synthesized from the RNA samples used for the microarray using oligo-dT primers. The qPCR reaction was performed using the LightCycler (Roche, Almere, NL) and the Fast Start DNA Master PLUS SYBRgreen 1 kit (Roche). Primer (Sigma Genosys, Cambridge, UK) sequences are listed in S1 File. Gene expression was calculated as normalized ratio and normalized to the housekeeping gene peptidylprolyl isomerase A (*Ppia*) [30]. All samples (NT *n* = 8, HT *n* = 8) were analyzed in duplicate and reported as mean ± standard error of the mean. To compare qPCR with microarray data, data were analyzed using one-tailed Student’s t-tests, with *P* < 0.05 considered significant.

### Hierarchical clustering analysis

A gene list with all significant differentially expressed genes at one hour, three days and fourteen days after HT was generated based on the microarray analysis. MeV open-source software [31] was used for the generation of heatmap and hierarchical clustering of genes and samples according to Pearson’s correlation of average linkage.

### Gene ontology (GO) analysis

For GO analysis, the significant gene list (*P* < 0.05, Fold change cutoff of log2(Fc) = < -0.125, or > 0.125) and a reference list comprising all unique genes (27,334, excluding pseudo- and predicted- genes) present on the microarray chip were imported into the software tool DAVID [32,33]. In DAVID a total of 11,838 unique genes, among the 27,334 probes, were mapped to the GO Panther database (www.pantherdb.org, version 9.0, release 20 January 2014). These were used to generate a reference gene list. Up- and down- regulated gene lists were mapped against the Panther database. Panther GO classes are greatly abbreviated and simplified to facilitate high-throughput analyses. Significant lists were statistically compared to the reference list to identify significant over-representation of genes (*P < 0*.*05*) within the GO classes for biological process. Classes with significant over-representation are shown and marked when, after correcting for multiple testing with BH-correction, *P* < 0.05.

### Radioactive (^33^P) *in situ* hybridization

To quantify the expression levels of *Nrxn1*, *Dpysl3*, *Ptprd*, and *Slit2* in the hippocampus, ^33^P *in situ* hybridization was performed on coronal sections (16 μm) from animals three days after eFS (NT *n* = 9, HT *n* = 9). ^33^P RNA probes were designed against the mRNA for *Nrxn1*, *Dpysl3*, *Ptprd*, and *Slit2* (*Nrxn1*; NM_020252.3 at 7176–7568 bp, *Dpysl3*; NM_009468.4 position at 1306–1914 bp, *Ptprd*; NM_011211.3 position at 4865–5415 bp, *Slit2*; NM_178804.3 position at 2152–2789 bp), and aligned to the region, which included the sequence of the significantly regulated microarray probe. Primer sequences are listed in S1 File. The synthesis of ^33^P RNA-probes and *in situ* hybridization were performed as described in previous work [34]. Briefly, sections were hybridized to the 1 x 10^6^ cpm of anti-sense ^33^P RNA probe in 150 μL hybridization mixture with 50% deionized formamide, 2x standard saline citrate (SSC), 10% dextrane sulphate, 1x Denhardt’s solution, 5 mM ethylenediaminetetraacetic acid (EDTA), and 10 mM phosphate buffer. After overnight hybridization of the probes at 72°C, slides were washed, dried and exposed film (Kodak Bio-Mac, MR). The developed films were digitized using an Epson flatbed scanner (Perfection 4990, Epson America, CA, USA). For each gene, one slide was hybridized to the sense control ^33^P RNA probe. No signal was observed in presence of the control sense ^33^P RNA probe (data not shown). Calibration dilutions of ^14^C micro-scales (Amersham Bioscience, Sweden) and anti-sense probes were used to determine the linear range for image quantification. An observer blind for treatment condition quantified photographs of the film in ImageJ. Regions of interest were placed in the subfields of the hippocampus bilaterally in 3 sections per animal. Expression levels were calculated by gray-scale value inversion, corrected for background gray-scale value and normalized. Mean intensity values of both hemispheres at 3 sections per animal were calculated and used for statistical analysis. Background value was determined within the corpus callosum. Bar graphs depict the average expression levels per condition and subfield, normalized to control (NT) group average with ± standard error of the mean. To compare expression levels of genes in HT versus NT animal couples Student’s t-tests (littermate-paired, two-tailed) were performed.

### Statistical analysis

All statistical analyses were performed using R with Stats package (version 3.1.2, http://cran.r-project.org). Thresholds of significance were set at α = 0.05.

## Results

### Regulation of neurofilament in the hippocampus after eFS

**Fig 1:**
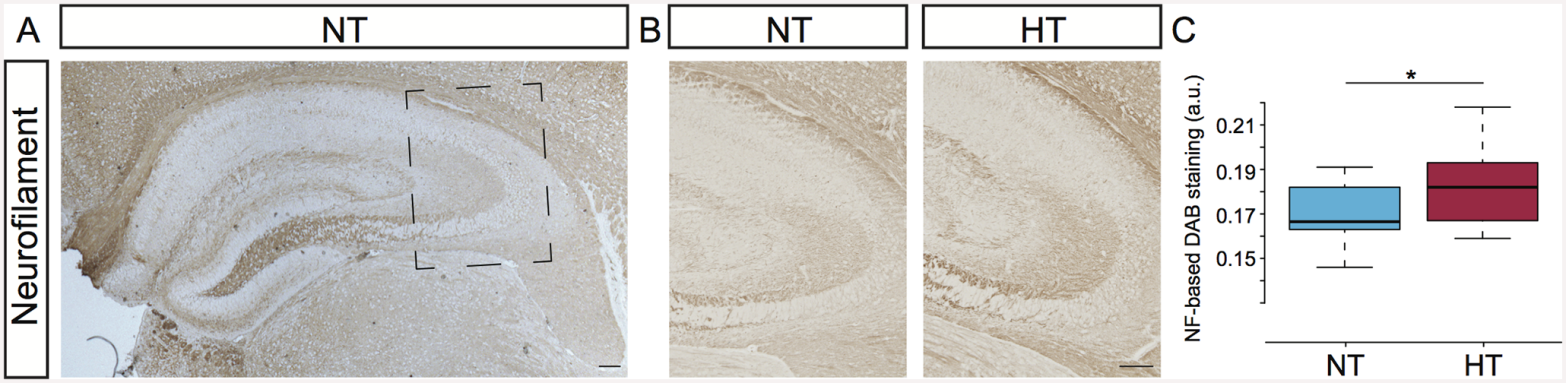
Hyperthermia induces upregulation of neurofilament protein-expression. (A) Neurofilament (NF) immunostaining in the hippocampus of a normothermic (NT) control animal, 14 days post-hyperthermia exposure. Square marks subfield enlargement; panel B. (B) Immunostaining for NF in the stratum lucidum of NT control animal (left panel) and in hyperthermia-exposed (HT) littermate (right panel). (C) Comparison of NF-based DAB staining in the stratum lucidum between NT and HT littermates. All HT (*n* = 10) animals showed increased DAB-staining in the stratum lucidum. Pair-wise comparison of DAB-staining intensities showed increased NF expression in HT animals compared to NT littermates (*n* = 10), Wilcoxon signed-rank test, * indicates a significance of *P* = 0.0049. Tukey-style box plots. Scalebars: 100 μm.

To explore the hypothesis that hyperthermia-induced eFS result in structural reorganization of the hippocampus, we compared neurofilament (NF, medium polypeptide) staining in hyperthermia-exposed mice (HT, *n* = 10) to normothermia-exposed control littermates (NT, *n* = 10) (Fig 1). NF is predominantly expressed in the neuropil of the hippocampus, such as the mossy fibers projecting from the DG to the CA3 region. HT exposed mice, compared to NT littermates, showed a significant increase in NF-staining in the stratum lucidum of the CA3 subfield (Wilcoxon signed-rank test *P* = 0.0049). NF-staining changes were specifically observed in the stratum lucidum and not in other subfields of the hippocampus. In line with published results in rats [15] our data show that eFS in mice trigger structural changes in the hippocampus.

### Regulation of hippocampal gene expression after eFS

To investigate which molecular pathways underlie the structural reorganization after eFS, we compared hippocampal mRNA content from HT and NT mice by microarray hybridization. Hippocampal tissue was sampled one hour, and three, fourteen, and fifty-six days after induction of eFS.

### Changes in mRNA expression within the hippocampus after eFS

One hour after eFS (NT *n* = 8, HT *n* = 8) 1004 differentially expressed probes were identified. Of these 1004 probes, 579 upregulated and 298 downregulated unique Ensembl annotated genes were identified (S2 File, excluding pseudo- and predicted-genes). The highest upregulated gene (8.04-fold), *Cyr61*, is an extracellular angiogenic factor. Brain *Cyr61* expression is induced by numerous stimuli. Although limited information is available on *Cyr61* function, it appears to be involved in vasculature or dendritic ramification of hippocampal neurons [35]. As expected, several highly upregulated genes are immediate early genes (IEGs, for example *Cyr61*, *Jun*, *Fos*, *Egr1*, and *Nr4a1*) and heat-shock proteins (*HSP110*, *Hspb1*, *Hsph1*, and *Dnajb1*) (Table 1). Amongst the highly downregulated genes trophic factor *Ptgds*, adhesion molecules *Pcdh20* and *Cldn5*, and transcription factors *Hes5* and *Sox18* were identified.

**Table 1:** Top 30 differentially expressed genes (up- and down- regulated) 1 hour after eFS.

1 hour post-eFS				
**Upregulated genes**				
**Ensembl**	**genesymbol**	**Description**	**Fc**	***P*-value BH-corrected**
ENSMUSG00000028195	*Cyr61*	cysteine rich protein 61	8.04	0.0000
ENSMUSG00000031558	*Slit2*	slit homolog 2 (Drosophila)	5.68	0.0000
ENSMUSG00000052684	*Jun*	Jun oncogene	3.13	0.0000
ENSMUSG00000021250	*Fos*	FBJ osteosarcoma oncogene	3.04	0.0000
ENSMUSG00000023034	*Nr4a1*	nuclear receptor subfamily 4, group A, member 1	2.99	0.0000
ENSMUSG00000038418	*Egr1*	early growth response 1	2.62	0.0000
ENSMUSG00000029657	*Hsph1*	heat shock 105kDa/110kDa protein 1	2.46	0.0000
ENSMUSG00000004951	*Hspb1*	heat shock protein 1	2.40	0.0000
ENSMUSG00000024190	*Dusp1*	dual specificity phosphatase 1	2.34	0.0000
ENSMUSG00000005483	*Dnajb1*	DnaJ (Hsp40) homolog, subfamily B, member 1	2.25	0.0000
ENSMUSG00000026628	*Atf3*	activating transcription factor 3	2.11	0.0000
ENSMUSG00000019997	*Ctgf*	connective tissue growth factor	2.08	0.0000
ENSMUSG00000022602	*Arc*	activity regulated cytoskeletal-associated protein	1.97	0.0000
ENSMUSG00000006057	*Atp5g1*	ATP synthase, H+ transporting, mitochondrial F0 complex, subunit c1 (subunit 9)	1.90	0.0000
ENSMUSG00000053877	*Srcap*	Snf2-related CREBBP activator protein	1.90	0.0000
ENSMUSG00000028214	*Gem*	GTP binding protein (gene overexpressed in skeletal muscle)	1.89	0.0000
ENSMUSG00000028907	*Utp11l*	UTP11-like, U3 small nucleolar ribonucleoprotein, (yeast)	1.86	0.0000
ENSMUSG00000020205	*Phlda1*	pleckstrin homology-like domain, family A, member 1	1.75	0.0000
ENSMUSG00000020818	*Mfsd11*	major facilitator superfamily domain containing 11	1.75	0.0000
ENSMUSG00000021810	*Ecd*	ecdysoneless homolog (Drosophila)	1.68	0.0000
ENSMUSG00000037266	*D4Wsu53e*	DNA segment, Chr 4, Wayne State University 53, expressed	1.65	0.0000
ENSMUSG00000020423	*Btg2*	B cell translocation gene 2, anti-proliferative	1.64	0.0000
ENSMUSG00000030847	*Bag3*	BCL2-associated athanogene 3	1.63	0.0000
ENSMUSG00000078698	*Mrgpra3*	MAS-related GPR, member A3	1.63	0.0000
ENSMUSG00000021453	*Gadd45g*	growth arrest and DNA-damage-inducible 45 gamma	1.62	0.0000
ENSMUSG00000031765	*Mt1*	metallothionein 1	1.61	0.0000
ENSMUSG00000021367	*Edn1*	endothelin 1	1.59	0.0000
ENSMUSG00000073676	*Hspe1*	heat shock protein 1 (chaperonin 10)	1.56	0.0000
ENSMUSG00000032531	*Amotl2*	angiomotin-like 2	1.55	0.0000
ENSMUSG00000048222	*Mfap1b*	microfibrillar-associated protein 1B	1.54	0.0000
**Downregulated genes**				
**Ensembl**	**genesymbol**	**Description**	**Fc**	**P-value BH-corrected**
ENSMUSG00000020863	*Luc7l3*	LUC7-like 3 (S. cerevisiae)	0.62	0.0000
ENSMUSG00000015090	*Ptgds*	prostaglandin D2 synthase (brain)	0.64	0.0000
ENSMUSG00000041378	*Cldn5*	claudin 5	0.68	0.0000
ENSMUSG00000071337	*Tia1*	cytotoxic granule-associated RNA binding protein 1	0.71	0.0000
ENSMUSG00000030495	*Slc7a10*	solute carrier family 7 (cationic amino acid transporter, y+ system), member 10	0.71	0.0000
ENSMUSG00000029570	*Lfng*	LFNG O-fucosylpeptide 3-beta-N-acetylglucosaminyltransferase	0.74	0.0000
ENSMUSG00000046470	*Sox18*	SRY-box containing gene 18	0.74	0.0000
ENSMUSG00000028645	*Slc2a1*	solute carrier family 2 (facilitated glucose transporter), member 1	0.74	0.0000
ENSMUSG00000000253	*Gmpr*	guanosine monophosphate reductase	0.77	0.0000
ENSMUSG00000030218	*Mgp*	matrix Gla protein	0.78	0.0047
ENSMUSG00000026822	*Lcn2*	lipocalin 2	0.78	0.0009
ENSMUSG00000030711	*Sult1a1*	sulfotransferase family 1A, phenol-preferring, member 1	0.79	0.0000
ENSMUSG00000040055	*Gjb6*	gap junction protein, beta 6	0.80	0.0000
ENSMUSG00000048001	*Hes5*	hairy and enhancer of split 5 (Drosophila)	0.81	0.0000
ENSMUSG00000046546	*Fam43a*	family with sequence similarity 43, member A	0.81	0.0000
ENSMUSG00000032422	*Snx14*	sorting nexin 14	0.81	0.0000
ENSMUSG00000029231	*Pdgfra*	platelet derived growth factor receptor, alpha polypeptide	0.81	0.0000
ENSMUSG00000050505	*Pcdh20*	protocadherin 20	0.81	0.0000
ENSMUSG00000022508	*Bcl6*	B cell leukemia/lymphoma 6	0.82	0.0000
ENSMUSG00000030707	*Coro1a*	coronin, actin binding protein 1A	0.82	0.0000
ENSMUSG00000041073	*Nacad*	NAC alpha domain containing	0.82	0.0000
ENSMUSG00000016921	*Srsf6*	serine/arginine-rich splicing factor 6	0.82	0.0000
ENSMUSG00000038175	*Mylip*	myosin regulatory light chain interacting protein	0.82	0.0000
ENSMUSG00000017760	*Ctsa*	cathepsin A	0.82	0.0000
ENSMUSG00000001240	*Ramp2*	receptor (calcitonin) activity modifying protein 2	0.82	0.0000
ENSMUSG00000043131	*Mob1a*	MOB kinase activator 1A	0.82	0.0000
ENSMUSG00000070167	*SNORA57*	Small nucleolar RNA SNORA57	0.83	0.0000
ENSMUSG00000075602	*Ly6a*	lymphocyte antigen 6 complex, locus A	0.83	0.0000
ENSMUSG00000028655	*Mfsd2a*	major facilitator superfamily domain containing 2A	0.83	0.0000
ENSMUSG00000050295	*Foxc1*	forkhead box C1	0.84	0.0000

Fc: Fold change represents expression values of HT animals divided by expression values of NT animals. *P*-value BH-corrected: Benjamini and Hochberg (BH) error corrected *p*-values.

**Fig 2:**
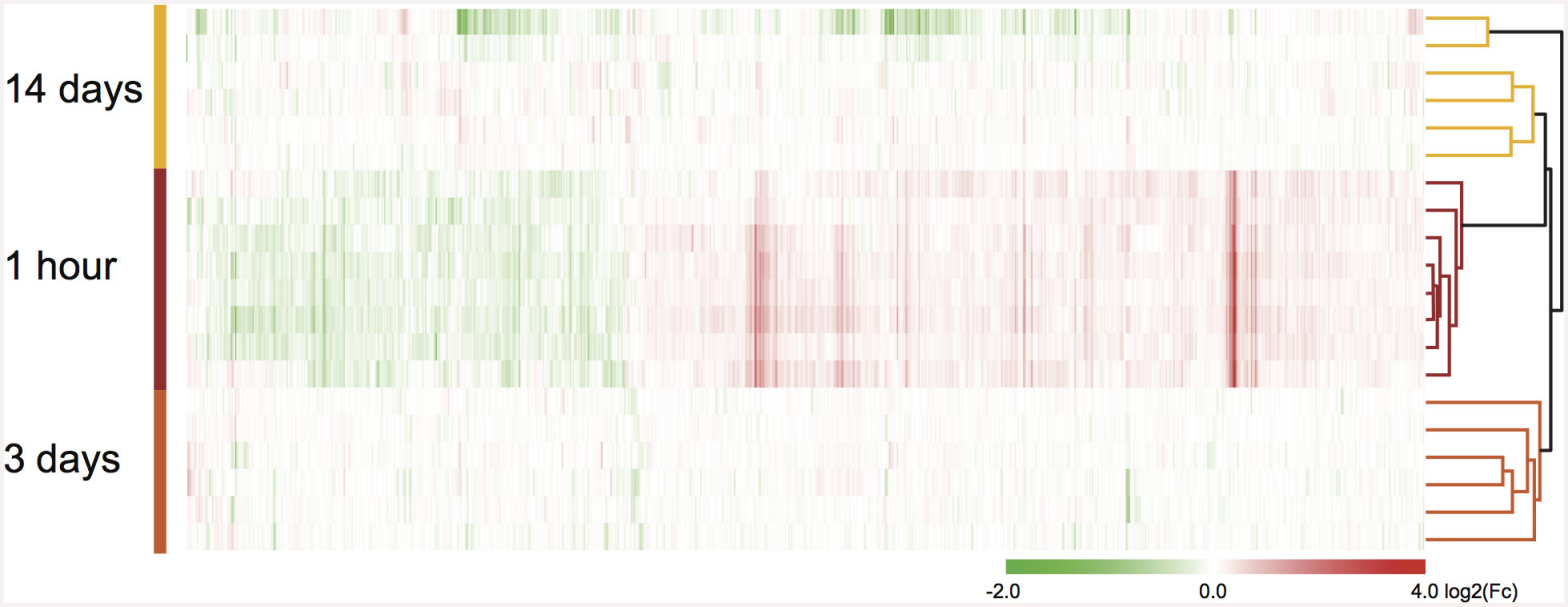
Hierarchical clustering analysis of mRNA expression after eFS. Hierarchical clustering analysis of differentially expressed genes (930) in all experimental littermate couples (1 hour, 3 and 14 days post-eFS). Rows depict differential expression levels per HT/NT littermate couple, per time-point after eFS. Clustering was performed based on gene and sample clustering using Pearson correlation on average linkage. Tree cluster, right from heat-map, shows hierarchical distance between samples from the different time points. Differential gene expression (log2(Fc) between HT/NT littermate couples is shown in the heatmap as upregulated (red), downregulated (green), or no change (white) according to colored-scale bar.

Analysis of the later time points identified 74, 15, and 1 significantly regulated probe(s) three, fourteen, and fifty-six days after eFS, respectively (Table 2, for all time points: NT *n* = 6, HT *n* = 6). Hierarchical cluster analysis performed on the total geneset of all differentially expressed genes from one hour, three and fourteen days after eFS (930 Ensembl annotated unique genes, excluding pseudo- and predicted- genes). Fifty-six days after eFS was excluded from the cluster analysis because only one significantly regulated gene was found. Differential expression levels (log2(Fc)) between each HT and NT littermate couple was used for clustering. Based on Pearson correlation of average linkage the HT samples and genes were clustered (Fig 2). Gene expression profiles of all samples showed strong clustering per time-point except for two samples (14 days post-eFS), which showed larger clustering distance to all other samples.

**Table 2:** Differentially expressed genes (up- and down- regulated) three, fourteen, and fifty-six days post-eFS.

**3 days post-eFS**				
**Upregulated genes**				
**Ensembl**	**genesymbol**	**Description**	**Fc**	**P-value BH-corrected**
ENSMUSG00000001911	*Nfix*	nuclear factor I/X	1.32	0.0414
ENSMUSG00000003410	*Elavl3*	ELAV (embryonic lethal, abnormal vision, Drosophila)-like 3 (Hu antigen C)	1.20	0.0319
ENSMUSG00000066551	*Hmgb1*	high mobility group box 1	1.16	0.0050
ENSMUSG00000071337	*Tia1*	cytotoxic granule-associated RNA binding protein 1	1.16	0.0195
ENSMUSG00000024501	*Dpysl3*	dihydropyrimidinase-like 3	1.16	0.0181
ENSMUSG00000024268	*Celf4*	CUGBP, Elav-like family member 4	1.14	0.0414
ENSMUSG00000001020	*S100a4*	S100 calcium binding protein A4	1.14	0.0317
ENSMUSG00000028399	*Ptprd*	protein tyrosine phosphatase, receptor type, D	1.14	0.0115
ENSMUSG00000024109	*Nrxn1*	neurexin I	1.13	0.0319
ENSMUSG00000038900	*Rpl12*	ribosomal protein L12	1.12	0.0022
ENSMUSG00000053128	*Rnf26*	ring finger protein 26	1.12	0.0252
ENSMUSG00000027962	*Vcam1*	vascular cell adhesion molecule 1	1.12	0.0319
ENSMUSG00000024799	*Tm7sf2*	transmembrane 7 superfamily member 2	1.11	0.0181
ENSMUSG00000003198	*Zfp959*	zinc finger protein 959	1.11	0.0312
ENSMUSG00000059325	*Hopx*	HOP homeobox	1.11	0.0181
ENSMUSG00000032047	*Acat1*	acetyl-Coenzyme A acetyltransferase 1	1.11	0.0119
ENSMUSG00000022194	*Pabpn1*	poly(A) binding protein, nuclear 1	1.11	0.0424
ENSMUSG00000060675	*Pla2g16*	phospholipase A2, group XVI	1.11	0.0331
ENSMUSG00000031451	*Gas6*	growth arrest specific 6	1.11	0.0414
ENSMUSG00000049932	*H2afx*	H2A histone family, member X	1.11	0.0119
ENSMUSG00000024411	*Aqp4*	aquaporin 4	1.11	0.0119
ENSMUSG00000030605	*Mfge8*	milk fat globule-EGF factor 8 protein	1.10	0.0195
**Downregulated genes**				
**Ensembl**	**genesymbol**	**Description**	**Fc**	**P-value BH-corrected**
ENSMUSG00000052305	*Hbb-b1*	hemoglobin, beta adult major chain	0.75	0.0002
ENSMUSG00000069919	*Hba-a1*	hemoglobin alpha, adult chain 1	0.80	0.0103
ENSMUSG00000069917	*Hba-a2*	hemoglobin alpha, adult chain 2	0.83	0.0195
ENSMUSG00000027375	*Mal*	myelin and lymphocyte protein, T cell differentiation protein	0.85	0.0012
ENSMUSG00000032517	*Mobp*	myelin-associated oligodendrocytic basic protein	0.86	0.0016
ENSMUSG00000006205	*Htra1*	HtrA serine peptidase 1	0.88	0.0103
ENSMUSG00000040055	*Gjb6*	gap junction protein, beta 6	0.88	0.0195
ENSMUSG00000006782	*Cnp*	2',3'-cyclic nucleotide 3' phosphodiesterase	0.89	0.0103
ENSMUSG00000049892	*Rasd1*	RAS, dexamethasone-induced 1	0.90	0.0119
ENSMUSG00000062591	*Tubb4a*	tubulin, beta 4A class IVA	0.90	0.0461
ENSMUSG00000070282	*3000002C10Rik*	RIKEN cDNA 3000002C10 gene	0.90	0.0195
ENSMUSG00000054459	*Vsnl1*	visinin-like 1	0.90	0.0423
ENSMUSG00000022594	*Lynx1*	Ly6/neurotoxin 1	0.90	0.0195
ENSMUSG00000036634	*Mag*	myelin-associated glycoprotein	0.90	0.0319
ENSMUSG00000079018	*Ly6c1*	lymphocyte antigen 6 complex, locus C1	0.91	0.0312
ENSMUSG00000090071	*Cdk5r2*	cyclin-dependent kinase 5, regulatory subunit 2 (p39)	0.91	0.0181
ENSMUSG00000017009	*Sdc4*	syndecan 4	0.91	0.0369
ENSMUSG00000028843	*Sh3bgrl3*	SH3 domain binding glutamic acid-rich protein-like 3	0.92	0.0119
**14 days post-eFS**				
**Upregulated genes**				
**Ensembl**	**genesymbol**	**Description**	**Fc**	**P-value BH-corrected**
ENSMUSG00000018501	*Ncor1*	nuclear receptor co-repressor 1	1.27	0.0494
ENSMUSG00000006782	*Cnp*	2',3'-cyclic nucleotide 3' phosphodiesterase	1.26	0.0295
ENSMUSG00000037625	*Cldn11*	claudin 11	1.25	0.0078
ENSMUSG00000028385	*Snx30*	sorting nexin family member 30	1.21	0.0112
ENSMUSG00000063077	*Kif1b*	kinesin family member 1B	1.18	0.0475
ENSMUSG00000025038	*Efhc2*	EF-hand domain (C-terminal) containing 2	1.17	0.0335
ENSMUSG00000029684	*Wasl*	Wiskott-Aldrich syndrome-like (human)	1.15	0.0112
ENSMUSG00000054459	*Vsnl1*	visinin-like 1	1.15	0.0494
ENSMUSG00000036526	*Card11*	caspase recruitment domain family, member 11	1.15	0.0123
ENSMUSG00000036985	*Zdhhc9*	zinc finger, DHHC domain containing 9	1.15	0.0475
ENSMUSG00000076439	*Mog*	myelin oligodendrocyte glycoprotein	1.13	0.0494
**Downregulated genes**				
**Ensembl**	**genesymbol**	**Description**	**Fc**	**P-value BH-corrected**
ENSMUSG00000023087	*Ccrn4l*	CCR4 carbon catabolite repression 4-like (S. cerevisiae)	0.86	0.0295
ENSMUSG00000030218	*Mgp*	matrix Gla protein	0.86	0.0037
**56 days post-eFS**				
**Downregulated genes**				
**Ensembl**	**genesymbol**	**Description**	**Fc**	**P-value BH-corrected**
ENSMUSG00000024617	*Camk2a*	calcium/calmodulin-dependent protein kinase II alpha	0.89	0.0120

Fc: Fold change represents expression values of HT animals divided by expression values of NT animals. *P*-value BH-corrected: Benjamini and Hochberg (BH) error corrected *p*-values.

**Fig 3:**
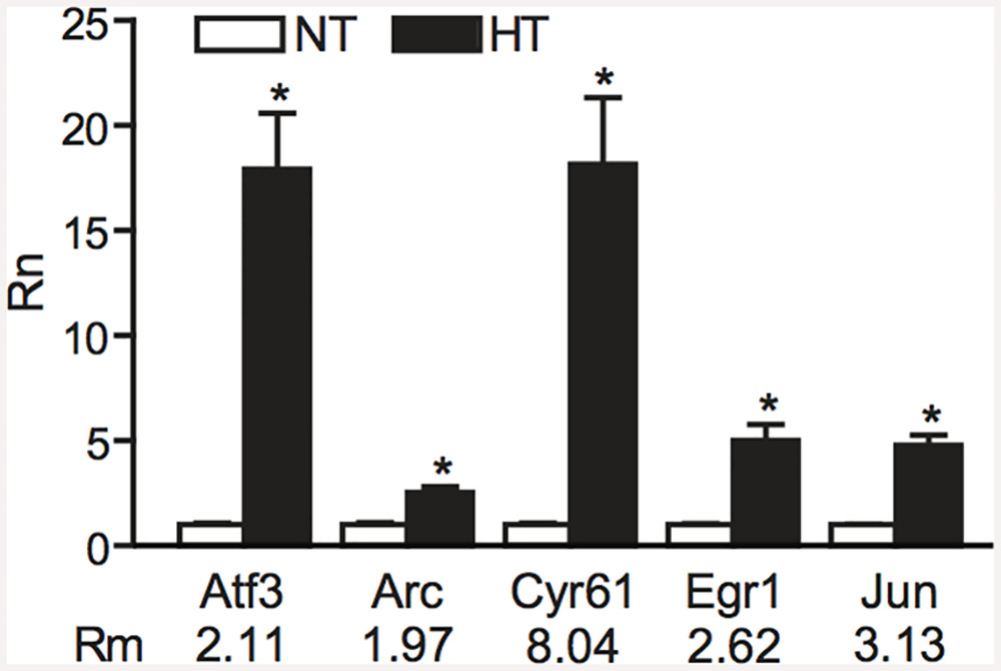
Validation of microarray results by qPCR analysis. Gene expression levels as determined by qPCR (Rn) in normothermic (NT) and hyperthermic (HT) animals compared to fold-change in expression of 5 genes as detected by microarray analysis (Rm). *P* < 0.05 was considered significant (one-tailed Student’s t-test). Indicated are mean ± standard error of the mean.

To independently validate the one hour after eFS microarray results, we used qPCR to quantify mRNA levels of several regulated genes in the HT/NT couples. *Jun*, *Cyr61*, *Atf3*, *Egr1*, and *Arc* were selected for validation (Fig 3). In line with the microarray data, all five genes were found to be upregulated in HT animals compared to NT littermates.

### Short- and long-term gene expression profile after eFS

To identify genes related to structural reorganization of the hippocampus we analyzed the gene sets three, fourteen, and fifty-six days after eFS. Compared to one hour after eFS, a limited number of genes was significantly regulated at three, fourteen, and fifty-six days after eFS (Table 2). Three days after eFS 22 upregulated and 18 downregulated unique genes were identified. In the 3 day gene list, several genes implicated in structural reorganization such as cell adhesion molecules (*Ptprd*, *Nrxn1*, *Dpysl3*, and *Vcam1*) were found to be upregulated, and myelin-sheath related genes (*Mobo*, *Cnp*, and *Mag*) downregulated. Interestingly, the top three downregulated genes were hemoglobin subunits, alpha (*Hba-a1 and Hba-a2)* and beta (*Hbb-b1*) chains. These three genes were upregulated at one hour after eFS. Information about the role of hemoglobin expression in the brain is limited [36]. Fourteen days after eFS 11 upregulated and 2 downregulated unique genes were identified. At this time-point, in contrast to the three days after eFS gene set, myelin-sheath related genes (*Cnp*, *Cldn11*, *Wasl*, and *Mog*) were upregulated. Analysis of fifty-six days after eFS samples revealed only 1 significantly downregulated gene (*Camk2a*).

### Gene ontology (GO) analysis of regulated genes after eFS

**Fig 4:**
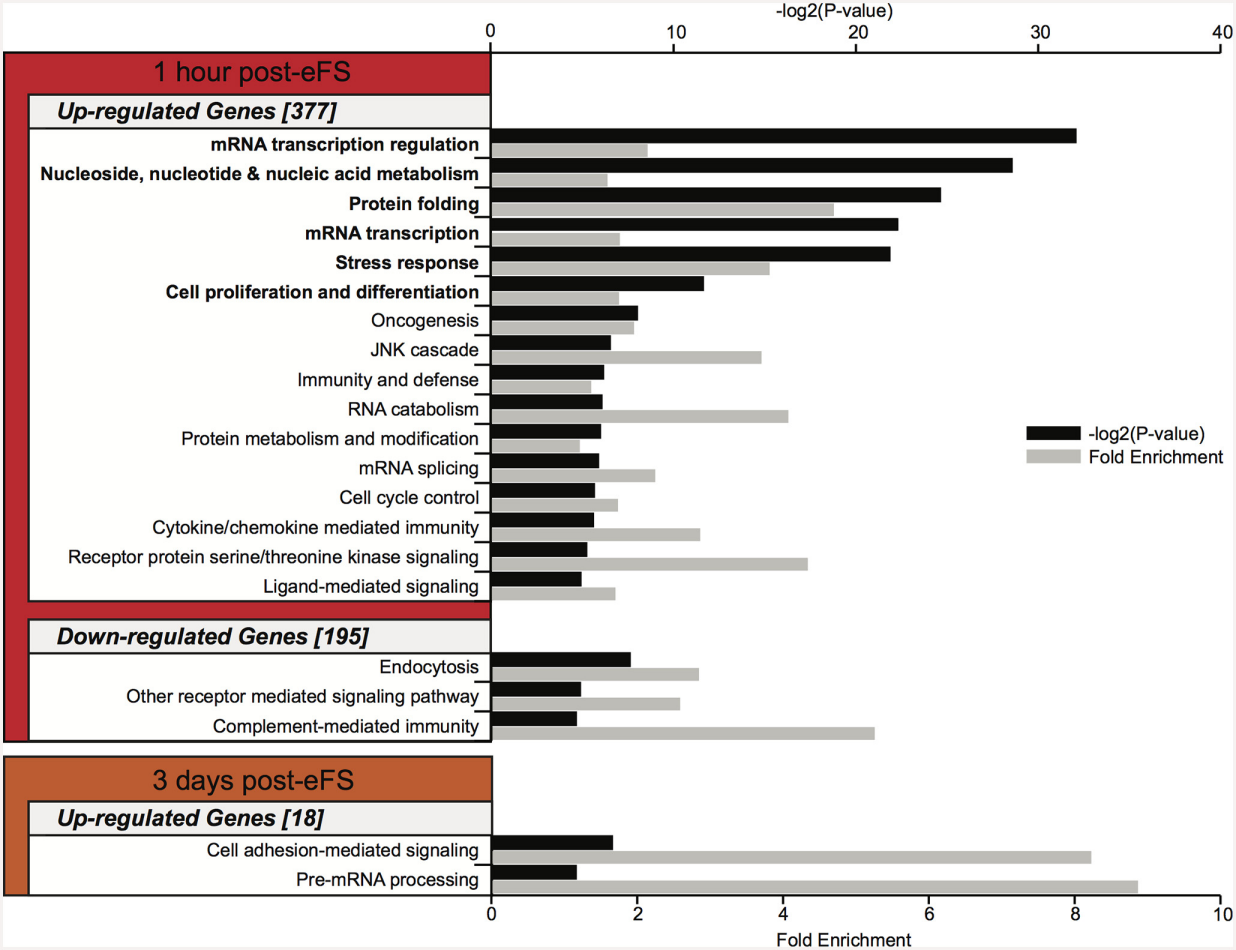
Gene ontology (GO) enrichment analysis on differentially expressed genes after hyperthermia exposure. GO biological process classifications with a *P* < 0.05 are depicted (up- and downregulation) per time point. Numbers between brackets represent total Panther GO annotated genes used for GO analysis per geneset. *P*-value per GO classification is shown (black bar, -log2(*P* value)), with corresponding fold enrichment (grey bar). Bold denoted GO classifications show significant enrichment (*P* < 0.05) after Benjamini and Hochberg (BH) error correction.

To identify common biological processes underlying changes after eFS we performed GO analysis using the DAVID web tool [32,37] based on GO Panther biological process (BP) annotation database (Mi 2012 Nucleic Acids Research). One hour after eFS upregulated genes significantly enriched GO biological process classifications such as mRNA transcription regulation, protein folding, stress response, and cell proliferation and differentiation (Fig 4, BH error corrected *P* < 0.05). Downregulated genes one hour after eFS were associated (but not significantly enriched after correction for multiple testing) to GO classification such as endocytosis and complement-mediated immunity. Three days after eFS upregulated genes were associated to GO classifications cell-mediated adhesion signaling and pre-mRNA processing (but did not reach significance after correction for multiple testing). Due to the low number of genes in the geneset no enrichment of GO classification was found within the downregulated genes at three days after eFS, nor within all regulated genes at fourteen and fifty-six days after eFS.

All together, the GO analysis reveals that within the genelists, specific biological processes can be identified 1 hour and 3 days after eFS. This urges us to identify candidate genes important after hyperthermia-induced eFS.

### Characterization of candidate genes implicated in structural reorganization after eFS

#### Transcriptional regulation at three days after eFS

Microarray gene expression analysis provided a list of significantly regulated genes three and fourteen days after eFS (Table 2). Closer examination of this list by GO analysis indeed showed transcriptional activity of genes related to structural reorganization. Based on literature and fold-change levels a number of candidate genes were selected. To characterize and validate the expression of these candidate genes we performed radioactive in-situ hybridization (RA-ISH) 3 days after eFS. ^33^P RNA-probes specific against *Nrxn1*, *Dpysl3*, *Ptprd*, and *Slit2* were used for analysis of mRNA expression in subfields of the hippocampus.

**Fig 5:**
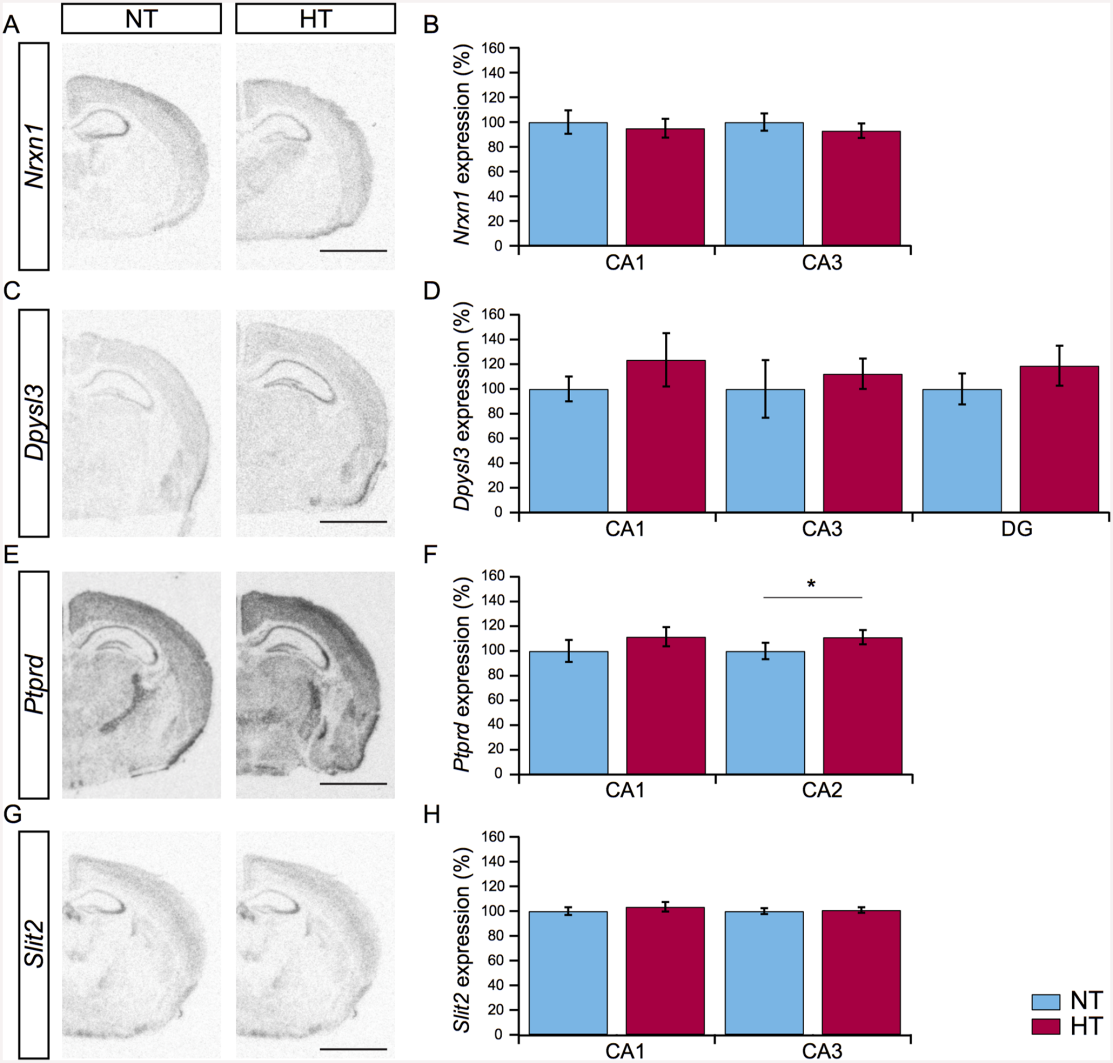
Expression of candidate genes mRNA related to structural reorganization. (A, C, E, G) Representative images of *Nrxn1*, *Dpysl3*, *Ptprd*, and *Slit2* gene expression in the hippocampus of NT (left panel) and HT (right panel) animals three days after eFS. (B, D, F, H) Comparison of *Nrxn1*, *Dpysl3*, *Ptprd*, and *Slit2* gene expression levels in subfields of the hippocampus in HT (*n* = 9, red bars) animals compared to NT (*n* = 9, blue bars) littermates. *Ptprd* is upregulated in the CA2 subfield of the hippocampus of HT animals, * indicates a significant difference of *P* = 0.0097, littermate-paired Student’s t-test. Bars represents mean ± standard error of the mean. Scalebars in panel A, C, E, and G: 500μm.

*Neurexin1* (*Nrxn1*) expression was prominent in the CA1 and CA3 subfields of the hippocampus. Nrxn1 is important during the formation of excitatory synapses, synaptogenesis [38] (Fig 5A). Quantification of *Nrxn1* showed no significant regulation of expression in HT animals within the CA1 and CA3 subfield (Fig 5B, NT *n* = 9, HT *n* = 9, littermate-paired Student’s t-test, *P* > 0.05). *Dpysl3*, also known as collapsin response mediator protein 4 (*Crmp4*), acts as a signaling molecule downstream from Sema3A, and is implicated in the regulation of neuronal dendrite development [39]. Expression of *Dpysl3* was quantified in the DG, CA1, and CA3 subfield of the hippocampus. A widespread increase in *Dpysl3* expression levels in all three subfields was observed (Fig 5C), but this increase was not statistically different between HT and NT littermates (Fig 5D, NT *n* = 9, HT *n* = 9, littermate-paired Student’s t-test, *P* > 0.05). *Ptprd*, Receptor-Type Tyrosine-Protein Phosphatase Delta, regulates axon guidance during brain development, and similar to *Nrxn1* is important during synaptogenesis [40]. Expression of *Ptprd* was observed in the CA1 and, remarkably, clearly present in the CA2 subfield known to be resistant to cell death in TLE patients (Fig 5E, [41]). Expression of *Ptprd* was significantly upregulated in CA2, but not in CA1, in HT animals compared to NT littermates (Fig 5F, NT *n* = 9, HT *n* = 9, littermate-paired Student’s t-test, CA2: *P* = 0.0097 CA1: *P* > 0.05). *Slit2* was included in the analysis, because it was the highest (5.68-fold) upregulated gene involved in structural reorganization, 1 hour after eFS. It regulates axon guidance during development of the hippocampus and is differentially regulated during experimental epileptogenesis and in temporal lobe epilepsy patients [42]. Pronounced expression of *Slit2* was observed in the CA3 subfield of the hippocampus (Fig 5G). No significant difference between HT and NT littermates was detected in relative expression levels 3 days after HT (Fig 5H, NT *n* = 9, HT *n* = 9, littermate-paired Student’s t-test, *P* > 0.05).

#### Myelin sheath-associated changes fourteen days after eFS

**Fig 6:**
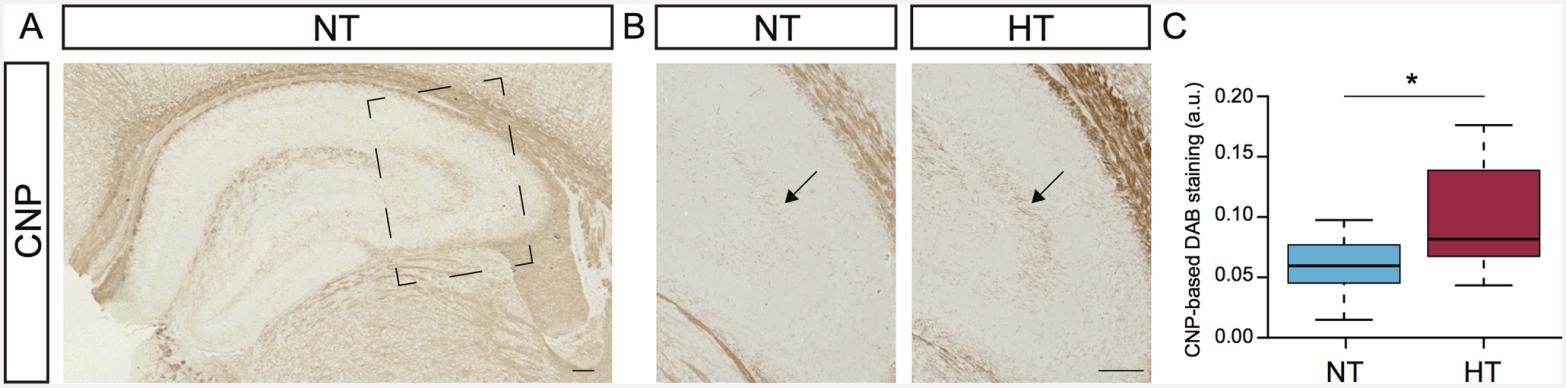
Hyperthermia induced upregulation of 2',3'-cyclic nucleotide 3' phosphodiesterase (CNP) in the stratum lucidum. (A) Normal expression of CNP in the hippocampus of a normothermic (NT) control animal 14 days post-hyperthermia exposure. Square marks subfield enlargement, panel B and C. (B) Expression of CNP in the stratum lucidum of NT control animal (left panel) and hyperthermia-exposed (HT) littermate (right panel). Arrows indicates CNP-positive staining in the stratum lucidum. (C) Comparison of CNP-based DAB staining in the stratum lucidum between NT and HT littermates. All HT animals showed increased DAB-staining in the stratum lucidum. Increased expression of CNP were observed in HT animals compared to NT littermates, Wilcoxon signed-rank test, * indicates *P* = 0.0078. Tukey-style box plots. Scalebars: 100μm.

The regulation of myelin-sheath associated genes three and fourteen days after eFS observed by the microarray gene expression analysis prompted us to monitor protein expression of 2',3'-cyclic nucleotide 3' phosphodiesterase (CNP) in hippocampus. This gene was downregulated 3 days after eFS (0.8-fold) and upregulated 14 days after eFS (1.26-fold). CNP is predominantly expressed in myelin sheaths and oligodendrocytes, although it’s physiological function remains ambiguous, loss of CNP affects the stability of axons [43–45]. Immunostaining of CNP protein at fourteen days after eFS revealed expression mostly in the neurophil of the hippocampus (Fig 6A). Interestingly, comparison of CNP expression between HT and NT littermates indicated elevated levels of CNP within the stratum lucidum of the CA3 region, specifically. Quantification and pair-wise comparison of CNP-based DAB staining in the stratum lucidum between HT and NT littermates showed pronounced upregulation of CNP in HT animals (Fig 6B and 6C, Wilcoxon signed-rank test *P* = 0.0078).

## Discussion

**Fig 7:**
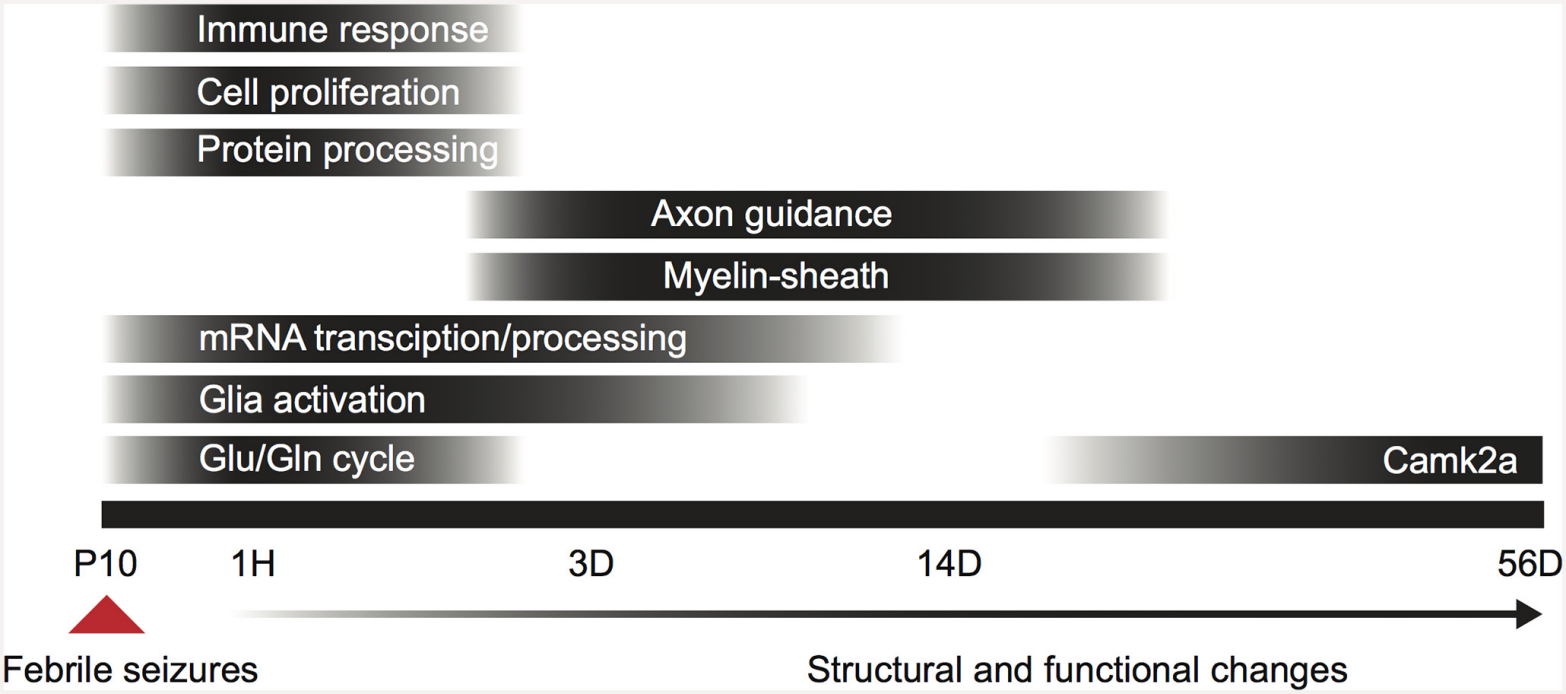
Timed regulation of biological processes after eFS. Bar blackness indicates the peak effect of the process. P10: postnatal day 10, 1H: 1 hour post-eFS, 3D: 3 days post-eFS, 14D: 14 days post-eFS, 56D: 56 days post-eFS, Glu/Gln cycle: glutamate/glutamine cycle.

In this study we investigated the short- and long-term changes in hippocampal gene expression following hyperthermia-induced eFS in mice. To pinpoint molecular mechanisms underlying structural reorganization after eFS, we performed a genome-wide transcriptome analysis at 4 time-points: immediately (one hour), shortly (three days) and during the late phase (fourteen and fifty-six days) after induction of eFS. At these time-points we identified 877, 40, 13 and 1 eFS regulated genes, respectively. Here we show that timed regulation of multiple processes, such as heat-, immune- and inflammation response, glutamate-glutamine cycle, myelination, and structural reorganization, are triggered by eFS (Fig 7). Fourteen days after eFS expression of neurofilament, a marker for dentate gyrus mossy fibers, and CNP, a marker for oligodendrocyte activation and myelination, was increased in the stratum lucidum of the hippocampus. This indicates that already 14 days after eFS structural reorganization is taking place (Fig 7). We provide evidence that specific processes and molecular candidates are involved in structural reorganization within the hippocampus at three and fourteen days after eFS. This is the first study to characterize the transcriptional response to eFS in mice throughout a time-course of 56 days.

### Transcriptional regulation after eFS

Analysis of hippocampal mRNA content identified 877, 40, 13, and 1 statistically significant regulated unique annotated gene(s) one hour, three, fourteen, and fifty-six days after eFS, respectively. Indicating a strong but transient regulation of gene expression during the first hour after eFS. As expected GO-analysis for gene enrichment related to specific biological processes reveals that eFS initially triggers a broad transcriptional response and a cellular response to heat (protein folding, stress response) as a direct response to the hyperthermia (Fig 7). Genes related to heat response included *HSP110*, *Hspb1*, *Hsph1*, and *Dnajb1*. Expression of IEGs (immediate early genes) such as, *Arc*, *Fos*, *Egr1*, *Atf1*, *Nr4a1*, *Cyr61*, *Jun* during and directly after eFS is most likely due to the prolonged seizure activity. Expression of these IEGs probably represents profound neuronal activity in the hippocampal network as demonstrated earlier by intra-hippocampal EEG recordings during and after eFS [12,16,46,47]. The lack of regulation of these genes 56 days after eFS is in line with the fact that at this time-point (the majority of) HT mice do not yet suffer from recurrent seizures. Comparing the genelists at the different time-points revealed that 35% of all regulated genes (14 out of 40 genes) three days after eFS were also regulated one hour after eFS. The majority of upregulated genes three days after eFS (5 out of 6 genes) showed downregulation 1 hour after eFS. In contrast, downregulated genes (5 out of 8 genes), with exception of the top-three regulated hemoglobin chain genes, 3 days after eFS were also downregulated 1 hour after eFS. Fourteen days after eFS, with exception of CNP, no overlap was observed with the regulated genes three days after eFS. However, 23% (3 out of 13 genes) of the regulated genes fourteen days after eFS did overlap with those 1 hour after eFS. Due to the low number of differentially expressed genes per time-point it is difficult to make over-time expression wave profiles per GO biological process [48,49]. The observed switch between one hour and three days after eFS from down- to upregulation of several genes indicates that over-time expression wave profiles can be observed after eFS.

#### Glutamate-glutamine cycle

As a consequence of increased hippocampal neuronal activity tight regulation of glutamate-glutamine cycle is of importance to avoid excitotoxic damage. Regulation of this process has already been described to play a key role in the susceptibility for febrile seizures and epileptogenesis in TLE patients [50–53]. Neuronal expressed glutaminase (Gls) converts glutamine to excitatory neurotransmitter glutamate. Glial glutamate transporters, such as excitatory amino acid transporter 2 (EAAT2, Slc1a2), avoid abnormal levels of extracellular released glutamate, by internalizing the neurotransmitter in the glial cells where it in turn is converted to glutamine by glutamine synthetase (Glul). One hour after eFS Glul is strongly reduced (0.90-fold), whereas Gls and Slc1a2 are upregulated (1.19-fold and 1.21-fold, respectively). The opposing regulation of Gls and Glul expression indicates an increase in glutamate production and less efficient synthesis of glutamine. As a consequence, changes in Glul and Gls expression may therefore contribute to an increase in neuronal excitability. Additionally, the upregulation of Slc1a2 might also reflect compensation to the increased glutamate production. Together with earlier findings of altered HCN-channel expression and function after eFS, the above-stated observations of Glul, Gls, and Slc1a2 expression indicate strong functional changes in neuronal excitability [17,21,54].

#### Immune and inflammatory response

Inflammatory processes, including regulation of chemokine and cytokine mediated immune response and complement-mediated immunity, are triggered during the first hour after eFS (Fig 7). Genes important in these processes included, *CCL-2*, *CCL-3*, *ILr10-a*/b, *C1qa*, and *Cr1l*. Febrile seizures in rats also show a robust immune response directly after exposure to HT [49,55]. Similarly, electrical kindling of the dentate gyrus as a model for TLE, showed overlap in biological processes with our genelists. This initial regulation of genes related to stress- immune- and inflammatory response among several epileptic animal models indicates that seizure activity, independent of the precipitating event, triggers immune response in the hippocampus [48]. A growing body of evidence shows involvement of immune and inflammatory processes in epilepsy and febrile seizures [25,56,57]. For example the role of interleukin-1ß (IL-1ß) in febrile seizures is a prominent topic of debate [5,58]. IL-1ß is a, pyrogenic, fever-promoting proinflammatory cytokine, which promotes the development of febrile seizures. Typically, loss of IL-1ß receptor in mice results in resistance to eFS [59]. We did not observe differential regulation of IL-1ß or its receptor, which suggests that regulation of hippocampal IL-1ß and its receptor is not important after eFS. However this does not exclude function of IL-1ß during eFS.

Based on our data, increased expression of chemokines CCL-2 (1.25-fold) and CCL-3 (1.17-fold) suggests regulation of blood-brain barrier permeability. Interestingly, both chemokines are found to be upregulated in TLE patients at mRNA transcript and protein level [25,57]. Expression of CCL-3 at the apical membrane of vascular endothelial cells triggers adhesion of leucocyte, whereas basal localization of CCL-2 to the membrane facilitates the migration of the leucocytes through the endothelial surface [60–62]. The regulation of chemokines therefore contribute to the permeability of the blood-brain barrier, which is associated with epileptogenesis [56]. In addition, both CCL-2 and CCL-3 trigger Ca^2+^ signaling in hippocampal neurons resulting in increased excitability and synaptic transmission [63,64].

Cytokine receptor IL-10ra and IL-10rb are differentially regulated 1 hour after eFS. The ligand, IL-10, is generally regarded to have anti-epileptogenic effects [65,66], and is upregulated in hippocampus and cortex of TLE patients [57]. IL-10 and its receptor are both upregulated as a consequence of excitotoxic injury [67]. This suggests that, as a compensatory mechanism, IL-10 expression is induced to avoid exacerbation of the neuronal damage. Interestingly, the alpha and beta subunit of the heterotetrameric IL-10 receptor are up- (1.13-fold) and downregulated (0.90-fold), respectively, after eFS. Unfortunately not much is known about the differences in characteristics of IL-10 receptor subunits, especially regarding the receptor function in the brain. Also little is known about the specific functional implications of the downregulated complement factors C1qa (0.88-fold) and Cr1l (0.91-fold) in epilepsy. Reduced expression of the complement factors may suggest suppression of the complement cascade. However, activation of the complement system, i.e. upregulation of complement mediators including C1qa and C1r, has been reported in both experimental and human temporal lobe epilepsy [68] and is thought to contribute to neuronal hyper-excitability and seizures [56]. In summary, our findings indicate that chemokines, cytokines and complement factors are regulated as a result of eFS.

### Genes involved in structural reorganization after eFS

Overrepresentation of genes to GO classification cell-mediated adhesion signaling indicates that structural reorganization seen 14 days after eFS is initiated 3 days after eFS (Fig 7). In this study we found significant upregulation of *Ptprd* within the CA2 region of the hippocampus 3 days after eFS. Ptprd localizes to the pre-synapse where it interacts with post-synaptic neurotrophin receptor tyrosine kinases (Trks) and bi-directionally activate signal transduction cascades to recruit synaptic proteins important for establishing and maintaining excitatory synapses [40]. Ablation of *Ptprd* may therefore result in altered CA1 synaptic transmission and learning-deficits [69]. The observed increase of *Ptprd* within the CA2 may therefore trigger changes at excitatory synapse projecting to the CA1 [70]. Multiple lines of evidence suggest regulation of class 3 semaphorins after kainate acid-induced epilepsy in rodents [71,72]. Dpysl3, a downstream effector of Sema3A, was significantly regulated 3 days after eFS in our microarray and its mRNA expression was found in all subfields of the hippocampus. However, the widespread increase of *Dpysl3* expression in HT animals did not reach significance. Validation of the candidate genes from the array, (*Dpysl3*, *Nrxn1*, and *Slit2*,) by in situ hybridization proved to be difficult. Most likely, this is due to heterogeneity in the transcriptional response of HT and NT littermates and detection sensitivity of RA-ISH.

Fourteen days after eFS, upregulation of CNP protein in the stratum lucidum confirmed our hypothesis that structural reorganization occurs after eFS. The upregulation of CNP indicates involvement of oligodendrocyte activation and myelin production. Interestingly, *CNP* mRNA expression is downregulated (0.89-fold) three days after eFS, similar to *Mobp*, *Mal*, and *Mag* (0.86, 0.85, and 0.90-fold, respectively), which all represent myelination and oligodendrocyte activation. However, fourteen days post HT *CNP* expression as well as myelin oligodendrocyte glycoprotein (*Mog*, 1.13-fold) is upregulated (1.26-fold). It would be interesting to learn what this switch in regulation of expression during the early to late phase after eFS means in terms of oligodendrocyte activation and myelination in the hippocampus. Possibly the reversal in expression has to do with different phases in structural reorganization, in which de- and re-myelination are required. It should be noted that mossy fibers to the CA3 are normally non-myelinated, hence the name stratum lucidum [73]. However, myelinated axons can be detected within the stratum lucidum, in particular after lesion of the enthorino-hippocampal perforant pathway [74]. This lesion induced axon sprouting in the stratum lucidum and recruited oligodendrocytes thought to myelinate the newly formed axon sprouts. Thus, our observed changes in CNP levels after eFS may indicate oligodendrocyte recruitment possibly contributing to myelination of newly sprouted axons. What the significance of these processes and oligodendrocytes is during epileptogenesis remains to be determined [75].

All together, our data suggest that proteins related to structural organization of the nervous system, such as cell adhesion molecules, axon guidance proteins, and myelin production are regulated after eFS. Kainate acid-induced experimental mesial temporal lobe epilepsy in rats trigger regulation of proteins involved in structural organization, axon guidance signaling pathways, 14 days post-induction [76]. The observed eFS induced changes in axon guidance and myelin-related genes, as well as the immune response activity mentioned earlier, might therefore resemble processes underlying epileptogenesis independent of the precipitating event.

### Glia activation

Interestingly, regulation of glia-associated genes was observed 1 hour and 3 days after eFS. *Gjb6* (*Connexin 30*), a gap junction protein expressed in glial cells, remained downregulated (0.80-fold) until 3 days after eFS. Gap junction proteins are important for cell-to-cell communication and exchange of small signaling molecules (<1000 Da) [77]. Other glia-associated proteins, such as *S100a4* and *Aqp4*, were upregulated 3 days after eFS (1.14- and 1.11-fold, respectively). *Aqp4* (aquaporin 4) is a water channel present at the astroglial endfeet contacting capillaries regulating transmembrane water transport [78]. Both *Aqp4* and *Gjb6* play important roles in regulation of osmolarity and energy metabolite housekeeping, therefore contribute to the epileptogenesis and are related to seizure severity [79]. Genetic ablation of either of the two genes results in prolongation of seizure duration or lowering of threshold for epileptic activity, respectively [79,80]. It remains to be determined by which mechanism these channels/junctions contribute to epileptogenesis. The regulation of these genes, including glia marker *S100a4*, indicates activation of glial cells shortly after eFS.

### Regulation of Camk2a 56 days after eFS

At 56 days after eFS, we found Camk2a to be the sole significantly (down)-regulated gene (Fig 7). Our results are in line with a recent study showing that Camk2a protein localization in the post-synaptic density (PSD) is reduced 25 days after recurrent febrile seizures [81]. In the same study, the phosphorylation state of Camk2a is changed in favor of Thr^305^ over Thr^286^ after recurrent febrile seizures. The reduction in Thr^286^ phosphorylation results in higher dissociation rates of Camk2a from the PSD [82]. Phosphorylation of Camk2a is a major trigger to consolidate synaptic potentiation during the early phase of long-term potentiation (LTP), an important mechanism for synaptic plasticity related to hippocampal learning [82]. The observed reduction in phosphorylation of Thr^286^ and presence of Camk2a in the spine by Xiong *et al*. [81] together with our observation of reduced Camk2a expression, may reflect a compensatory mechanism as a consequence to increased neuronal activity. Since lower number of and phosphorylation of Camk2a (Thr^286^), would result in decreased probability to elicit LTP at the synapse. Transcriptome analysis in the CA3 after electrical kindling of the DG also showed marked reduction of Camk2a during the latent phase, in addition to reduction directly and 1 week after kindling [48]. Our data support the notion that regulation of CaMK2a is involved in epileptogenesis.

### Heterogeneity in transcriptional response during late phase after eFS in mice

As mentioned earlier, in rat studies about 35–68% of all eFS animals show spontaneous epileptiform activity [15,22,23], which indicates that there is heterogeneity in response to eFS. All individual samples taken one hour, three days and fourteen days after eFS were used for hierarchical clustering analysis and identified three clusters based on their transcription profiles. All samples within each cluster belonged to the same time-point. This indicates that transcriptional profiles of all individuals per time-point show a high degree of similarity, especially during the early phase after eFS. However, fourteen days after eFS sample cluster behavior shows a certain degree of heterogeneity in transcriptional response. This heterogeneity may reflect the process of epileptogenesis and its relation to susceptibility to develop spontaneous recurrent seizures. Because of the study design we cannot correlate transcriptional profiles with spontaneous recurrent seizure outcome. Further longitudinal studies in mice will be required to determine which animals develop spontaneous recurrent seizures after eFS and whether changes in transcriptome profiles can be used to predict seizure outcome.

## Concluding Remarks

The eFS solely may not be responsible for initiation of epileptogenesis but contribute to its process. The observed changes in structural organization and expression of genes related to this process confirm that eFS induce changes in the brain. These changes most likely contribute to or occur simultaneously with functional changes, such as HCN-channel conductance, altered GABAergic function, increased neuronal excitability, and shifted activity-dependent synaptic plasticity, described in other studies related to febrile seizures [14,18,47,54,83]. Other precipitating and susceptibility factors, such as stress [84] or genetic background [10,11], in concert with prolonged eFS, are likely to contribute to the development of TLE. It is of interest to study which factors increase the chance of spontaneous recurrent seizures later in life. The combination of these factors with prolonged eFS, or repetitive exposure to eFS, will be required to validate our findings and provide valuable insight in the molecular mechanism behind the process of epileptogenesis. The observed local changes in NF and CNP expression indicate that hippocampal subfield transcriptome analysis after eFS might contribute to the identification of candidate genes. Additionally, identification of markers for epileptogenesis would greatly facilitate the ability to identify mice that will develop spontaneous recurrent seizures. This is the first study to identify regulated genes during a time-course of 56 days after eFS in mice (Fig 7). Our findings, in support with previous studies, identified initial regulation of immune and inflammation responses and glutamate-glutamine cycle. During the early phase and late phase glial-, myelin-, and axon guidance/cell adhesion related genes are regulated, together with increased expression of NF and CNP. These latter findings point to structural reorganization of the hippocampus after eFS in mice.

## Supporting Information

S1 File Sequences of primers used for qPCR and ^33^P RNA probe generation. Table lists all primers used for qPCR experiments and the generation of ^33^P RNA probes for radioactive *in situ* hybridization.
(XLS)

S2 File All differentially expressed genes (up- and down- regulated) 1 hour after eFS. Fc: Fold change represents expression values of HT animals divided by expression values of NT animals. *P*-value BH-corrected: Benjamini and Hochberg (BH) error corrected *p*-values.
(XLS)
